# Gut Mucosal Microbiome Signatures of Colorectal Cancer Differ According to BMI Status

**DOI:** 10.3389/fmed.2021.800566

**Published:** 2022-02-08

**Authors:** Sophie Shaw, Susan Berry, John Thomson, Graeme I. Murray, Emad El-Omar, Georgina L. Hold

**Affiliations:** School of Medicine, Medical Sciences and Nutrition, University of Aberdeen, Aberdeen, United Kingdom

**Keywords:** colorectal cancer, gut microbiota, high throughput sequencing, increased body mass index, colonic mucosa

## Abstract

**Background:**

Carrying excess body weight is a strong risk factor for colorectal cancer (CRC) development with ~11% of CRC cases in Europe linked to being overweight. The mechanisms through which excess body weight influences CRC development are not well understood but studies suggest the involvement of the presence of chronic low-grade inflammation and changes in the gut microbiota are involved.

**Aim:**

To compare the mucosal associated microbiota of patients with CRC to understand whether carrying excess body weight was associated with a unique CRC microbial signature.

**Methods:**

Microbiota signatures from colonic mucosal biopsies of CRC lesions and adjacent normal mucosal samples from 20 patients with overt CRC were compared with 11 healthy controls to see if having a BMI of >25 kg/m^2^ influenced colonic microbial composition.

**Results:**

Colonic mucosa samples from patients with CRC confirmed previously reported over-abundance of Fusobacteria associated with CRC but also an increase in Fusobacteria and *Prevotella* were associated with a BMI of >25 kg/m^2^. Correlation analysis of bacterial taxa indicated co-exclusive relationships were more common in CRC patients with a BMI >25 kg/m^2^ with an increase in transphylum relationships also seen in this patient group.

**Conclusions:**

The findings suggest that gut microbiota composition in patients with CRC is influenced by BMI status. Further understanding/defining these differences will provide valuable information in terms of developing novel pre-onset screening and providing post-manifestation therapeutic intervention.

## Introduction

Colorectal cancer (CRC) is the second leading cause of cancer-related deaths globally, with ~860,000 recorded deaths per year (1). CRC incidence is rising in parallel with the proportion of people carrying excess body weight (2, 3). Whilst several genetic factors have been shown to have an aetiologic role in CRC (4), the majority of sporadic CRC is largely attributable to environmental factors, such as obesity, smoking, and dietary factors (5–7). Being overweight (BMI > 25 kg/m^2^) is a well-known risk factor for cardiovascular disease and metabolic disorders, such as diabetes (8–10). However, a growing number of epidemiological studies show that carrying excess body weight, in the form of body fat, is associated with an increased risk of cancer, such as CRC (3, 11). As the global prevalence of obesity continues to rise, this will potentially lead to a further increase in the global incidence of CRC.

Suggested mechanisms linking excess body weight and CRC risk include the chronic low-grade inflammation which is associated with both conditions (12–15). It is commonly accepted that the gut microbiota strongly influences host health (16) and there is growing evidence to show that the gut microbiota is able to initiate inflammation as well as being linked to excess body weight and CRC development (17–20), with the gut microbiota known to be influenced by many factors, such as diet, environmental exposures, genetics, health status, and lifestyle (16, 17, 21, 22). We set out to assess whether carrying excess body weight was associated with a unique microbial signature in CRC. We performed 16S rRNA gene sequencing on paired colonic mucosal biopsies (adjacent normal mucosa and CRC tissue) from patients undergoing surgical resection for CRC and compared the findings to microbial signatures from colonic mucosal biopsies from healthy individuals. We focussed on identifying distinct taxonomic configurations as well as exploring co-occurrence networks.

## Methods

### Subject Recruitment

Study participants were recruited from subjects who had presented for the screening colonoscopy as part of the national colorectal cancer screening or patients undergoing colonic resection for CRC. Samples from colonoscopy patients were collected from 11 patients who had no colonic microscopic or macroscopic pathology (subsequently referred to as healthy subjects). All participants were from the same demographic as the patients with adenoma and CRC, and all had undergone a similar bowel cleansing procedure. No subjects had taken antibiotics in the 6 months prior to sampling. All samples were taken from the sigmoid colon. Participants were stratified based on body mass index (BMI) and classified as Healthy Weight (BMI of 20–25 kg/m^2^) or Overweight (BMI > 25 kg/m^2^). Sequence data from the healthy subjects were published previously and re-analysed within this study (23). All participants were from the Scottish Colorectal Cancer Screening Program, who had been invited to attend for colonoscopy following a positive faecal occult blood test (24). No colonoscopy participants had received antibiotics for 6 months prior to their endoscopy procedure. Surgical resection samples were collected from twenty-eight patients and none of them had received pre-operative therapy and all had the tumour surgically excised.

### Sample Collection

Biopsies were collected during colonoscopy using standard endoscopic forceps (Boston Scientific Nanterre Cedex France). Pinch biopsies were either fixed for histological assessment or placed directly into a 1.5 ml Eppendorf tube and snap-frozen in liquid nitrogen and transferred to a −80°C freezer until further analysis; within 1 month. All surgical resection samples were provided by the Grampian Biorepository who provided snap frozen tissue from both normal and tumour.

### DNA Extraction

Genomic DNA was extracted from colonic samples using the QIAamp DNA Mini Kit (Qiagen, Crawley, UK) using minor modifications of the manufacturer's instructions. Biopsy samples were kept frozen until the addition of ATL buffer before allowing biopsies to equilibrate to room temperature, an additional 10 μl of Proteinase K was added for an initial lysis period of 18 h to ensure complete lysis of the biopsy material prior to the DNA extraction (25). A series of blank samples were included which comprised DNA extraction kit reagent blanks as well as sterile water blanks.

### PCR Amplification and Sequencing

All participant samples and blanks were subjected to 16S rRNA gene sequencing. The V3-V4 region of the 16S rRNA gene was amplified using BAKT_341F (CCTACGGGNGGCWGCAG) and BAKT_805R (GACTACHVGGGTATCTAATCC) primers. The primers were designed with the Illumina adapter overhang already included. Amplification was performed using the Q5 polymerase kit following the instructions of manufacturer (New England Bio, Ipswich, MA, USA). Post-amplification, samples were purified using AMPure XP (Beckman Coulter, Brea, CA, USA) according to protocols of manufacturer. The samples were then indexed using the Nextera XT Index Kit V2 (Illumina, San Diego, CA, USA) and KAPA HiFi Hotstart ReadyMix (Kapa Biosystems, Cape Town, South Africa) with a short cycle PCR step followed by a clean-up with AMPure XP. The libraries were quantified using Quant-iT™ dsDNA Assay Kit HS (Thermo Fisher Scientific, Waltham, MA, USA) and analysed on a FLUOstar Omega plate reader (BMG LABTECH, Ortenberg, Germany). The library size was determined using the Agilent 2200 TapeStation (Agilent Technologies, Santa Clara, CA, USA). The libraries were pooled at equimolar concentrations in preparation for sequencing. Sample sequencing was performed using an Illumina MiSeq sequencer (Illumina, San Diego, CA, USA) using Illumina V3 chemistry and paired-end 2 × 300 base pair reads by the Centre for Genome Enabled Biology and Medicine, University of Aberdeen.

### Bioinformatics Analysis

QIIME version 1.9.0 (26) was used to merge paired end reads, quality philtre, pick open reference operational taxonomic units (OTUs) against the GreenGenes 13.8 database (27, 28) based on a 97% similarity, align representative sequences, remove singleton OTUs, and assign taxonomy. A series of blank samples were included from DNA extraction through library preparation and sequencing. Blank samples had a total of 278 sequences, equating to 70 ± 8 (mean ± SEM) reads per blank sample. This number was sufficiently low enough for us to determine that the contamination of samples had not occurred during the library preparation and sequencing.

For all sample cohorts, diversity was assessed using QIIME. Alpha and Beta diversity metrics; Observed OTUs, Chao (29), Shannon (30), Simpson (31), Good's Coverage, Bray Curtis (32), and Jaccard (33) were calculated using a subsampling depth of 3,689 sequences per sample. Rarefaction curves demonstrated that this subsampling level was sufficient to capture ample sample diversity (Supplementary Figure 1). Community structures were compared using the principal coordinates analysis (PCoA) plots generated using the Bray Curtis distance metrics and visualised using Emperor. Linear discriminant analysis effect size (LEfSe) analysis (34) was carried out for the identification of discriminative biomarkers associated with meta-data categories. Statistical analysis of stratification by metadata category was performed using PERMANOVA *via* the compare_categories script of QIIME using the Adonis function with 999 permutations. Subsequent statistical analysis was done in R 3.2.2 (35). Differential taxonomic abundance testing of the healthy and CRC sample set and figure generation was performed by converting the OTU table to a PhyloSeq object (36) and testing for changes in abundance using DESeq2 (37). Heatmaps were produced using the heatmap.plus package for R. All other figures were created using the ggplot2 package for R. Colours palettes from the R package RColorBrewer were used within plots.

### Co-occurrence Analysis

Rarefied taxa abundances at the species level were used to calculate the co-occurrence metrics for the CRC sample set using SparCC (38). Within group taxon-taxon correlation coefficients were calculated as an average of 20 inference iterations and pseudo *p* were calculated using 1,000 permuted datasets. The values of *p* were corrected for multiple testing using the Benjamini–Hochberg method. Taxon-taxon correlations >0.6 and < −0.6 with an adjusted *p* < 0.05 were visualised using Cytoscape.

## Results

### Mucosal Microbial Communities Are Affected by BMI Status as Well as the Adenoma-Carcinoma Sequence

The 16S rRNA gene sequence data were processed using TrimGalore! to remove the primer sequences. Within the CRC cohort, the total number of raw paired read sets was 27,536,992 with a mean number of sets of paired reads per sample of 162,584 (Supplementary Table 1A). The healthy sample 16S rRNA gene sequence data had a total of 1,019,169 raw paired read sets, with an average of 92,651.73 paired reads per sample (Supplementary Table 1B). After trimming with TrimGalore!, CRC samples had a mean number of paired reads of 60,389 and the healthy samples had a mean number of paired reads of 85,714.55.

To determine associations of colonic microbiome profiles with BMI status, we performed 16S rRNA gene sequencing on subjects with CRC. We compared the microbiome profiles with sequence data from healthy subjects who had attended for CRC screening on the basis of a positive faecal occult blood test but were subsequently confirmed to have no macroscopic or microscopic evidence of colonic disease (Tables 1A,B) (23). For subjects with CRC, tissue was available from both the tumour and adjacent normal mucosa. Tissue samples from the 11 healthy subjects were collected from the sigmoid colon as the majority of CRC and adjacent normal mucosa samples were from the distal colon and previous studies have confirmed that there are limited differences in the microbial diversity across the colon. We stratified subjects into 2 groups based on the BMI status: (1) Healthy weight with a BMI of 20–25 kg/m^2^, and (2) Overweight with a BMI of >25 kg/m^2^. The effect of BMI on alpha diversity was assessed, across all sample types—normal mucosa (healthy subjects), normal mucosa (CRC patients), or CRC tissue based on OTU richness, diversity, and evenness. We studied the healthy subjects comparing alpha diversity based on the BMI status. The CRC patient groups were initially stratified by (a) tissue type (lesion vs. adjacent normal mucosa) or (b) BMI status and then further analysed to encompass both tissue type and BMI status. No differences in the alpha diversity were seen across any comparisons made (Supplementary Figures 2, 3; *p* > 0.05, for all analyses, Wilcoxon rank test). In addition, we assessed alpha diversity between normal mucosa samples from the healthy subjects compared with CRC patients. A marginal but non-significant decrease in the alpha diversity was observed in the normal mucosa samples of CRC patient compared with healthy subjects (Supplementary Figure 4, *p* > 0.05, for all analyses, Wilcoxon rank test), demonstrating that bacterial richness in normal mucosa was similar between healthy subjects and patients with CRC. Further stratification by BMI status failed to demonstrate differences suggesting that there was comparable community evenness between the subject groups (Supplementary Figure 4, normal weight individuals *p* = 0.440, overweight individuals *p* = 0.181, Wilcoxon rank test of observed species).

**Table 1:** Study cohort information for patients with colorectal cancer.

**CRC patient cohort**	**All subjects**	**Sequencing cohort**
Number of patients (*N*)	28	20
Gender (% M:F)	39:61	35:65
Average age, in years, at procedure (range)	72 (47, 88)	71.5 (47, 87)
BMI status (kg/m^2^)		
20–25 >25	12 16	10 10
**Sample location (%)**		
Caecum Ascending colon Transverse colon Splenic flexure Sigmoid colon Rectum	25 17.9 10.7 3.6 35.6 7.2	30 5 25 15 5 20
Extramural venous invasion (% yes:no)	39:61	35:65
**Dukes stage (%)**		
*A* *B* *C* (C1:C2)	7 57 36 (32:4)	5 55 40 (35:5)
**TNM staging (%)**		
T2N0 T3N0 T3N1 T4N0 T4N1 T4N2	2 (7%) 13 (46%) 6 (21%) 3 (10.5%) 2 (7%) 1 (3.5)	1 (5%) 8 (40%) 6 (30%) 3 (15%) 1 (5%) 1 (5%)

*Participants were stratified based on body mass index (BMI) and classified as Healthy Weight (BMI of 20–25 kg/m^2^) or Overweight (BMI > 25 kg/m^2^). Eight of the microbial profiles of patients with CRC were discarded due to low sequencing depth in either the CRC or adjacent normal mucosa sample*.

**Table 2:** Study cohort information for healthy patients.

**Healthy patient cohort metadata**	
Number of patients (*N*)	11
Gender (% M:F)	9:91
Average age, in years, at procedure (range)	58 (52, 67)
**BMI status (kg/m** ^**2**^ **)**	
20–25 >25	5 6
**Sample location (%)**	
Sigmoid colon	100

**Figure 1:**
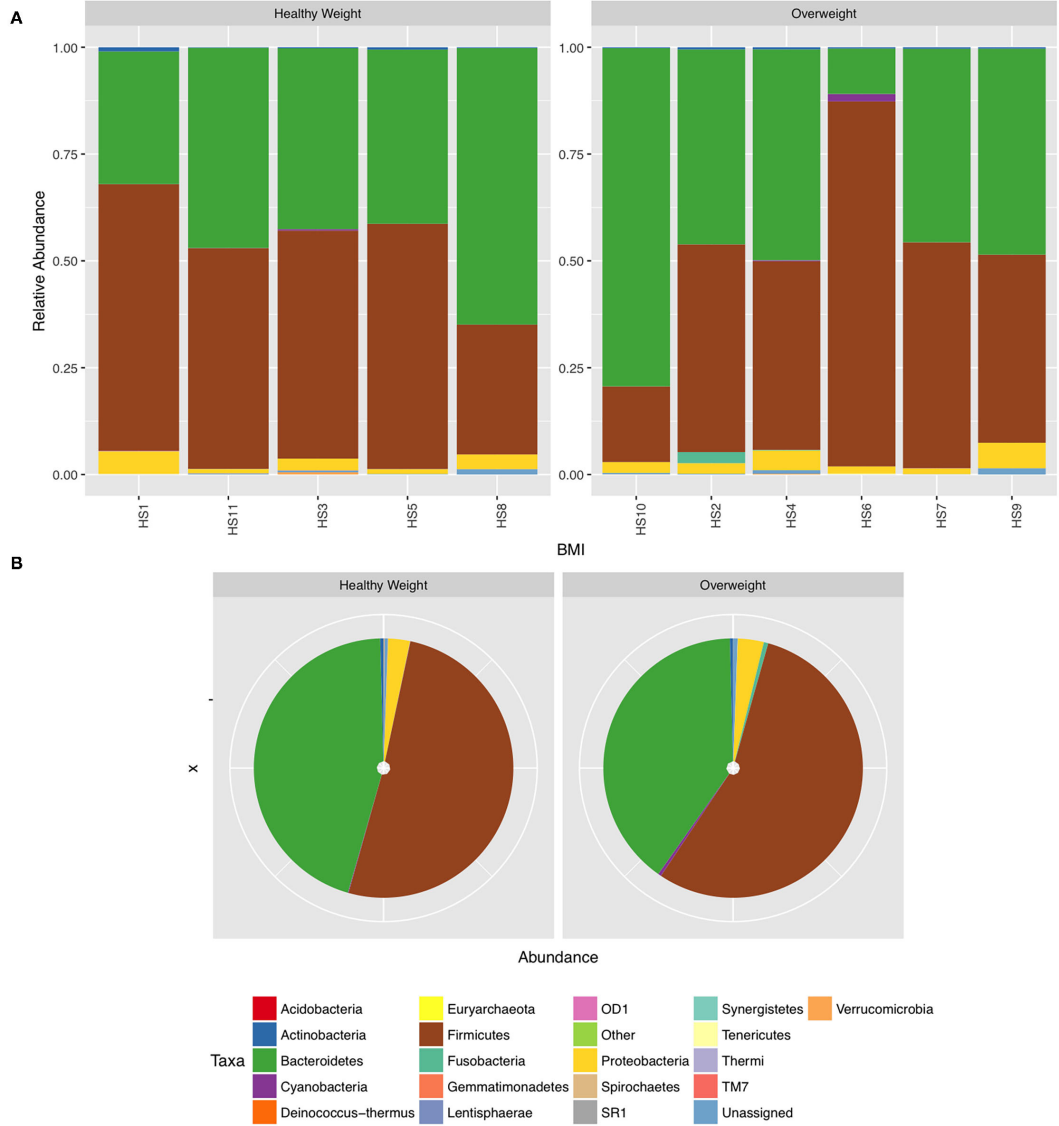
Relative abundance at phylum level for healthy samples stratified by body mass index (BMI). **(A)** Individual subject stacked bar charts. **(B)** Collective pie chart representation.

**Figure 2:**
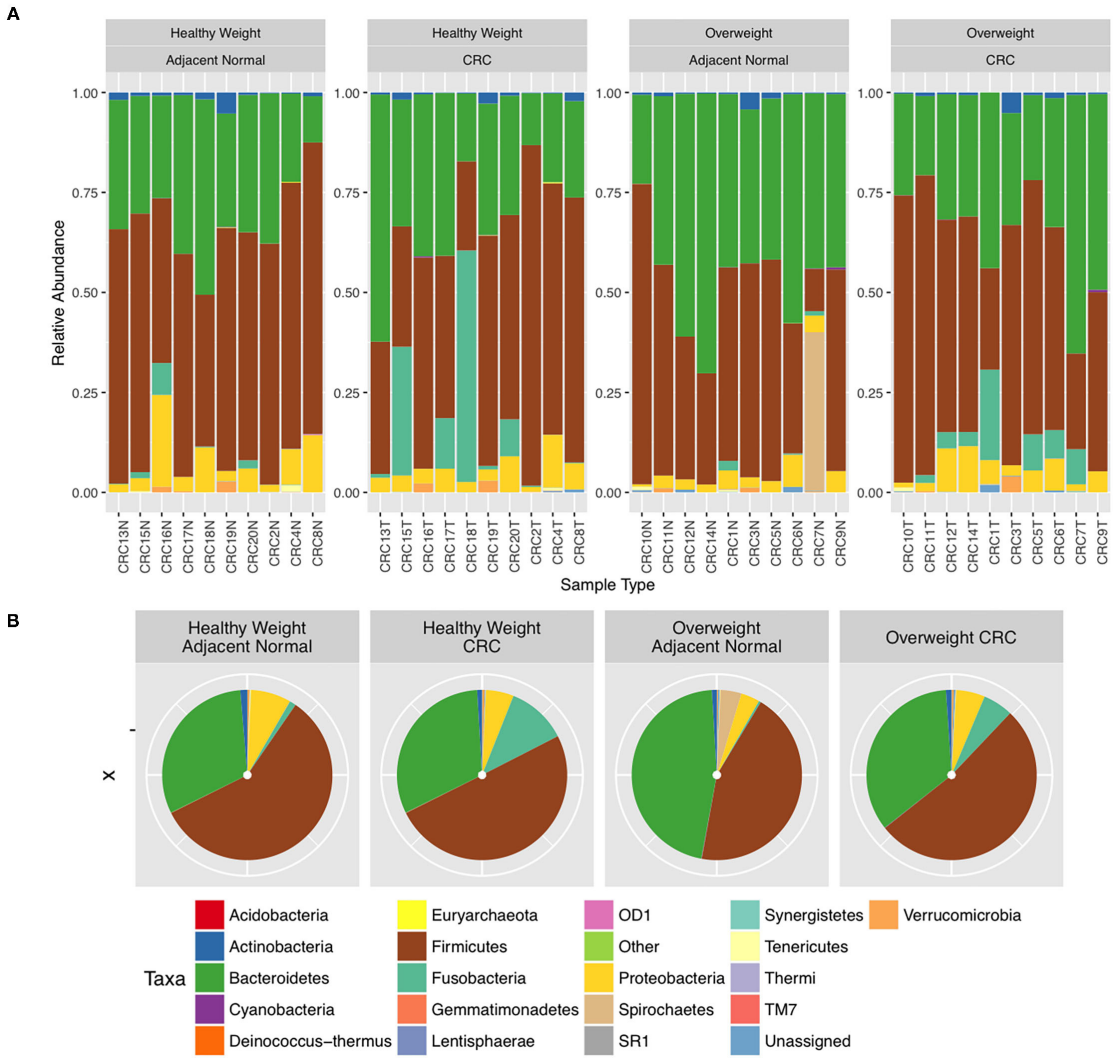
Relative abundance at phylum level for colorectal cancer (CRC) tissue and paired adjacent normal mucosa samples. **(A)** Individual subject stacked bar charts. **(B)** Collective pie chart representation.

We next conducted relative abundance analysis which indicated that the dominant phyla between the 2 subject groups varied. Firmicutes was the dominant phyla in healthy subjects (median 51.66%; interquartile range [IQR] 44.09%, 55.36%); followed by Bacteroidetes (median 45.61%; IQR 41.54%, 48.76%); Proteobacteria (median 2.54%; IQR 1.50%, 4.03%); and Actinobacteria (median 0.34%; IQR 0.23%, 0.51%; Figure 1; Table 2; Supplementary Figure 5). This profile was independent of BMI status. When a similar comparison was undertaken for CRC patient samples, there was a shift in the dominant phyla with Fusobacteria replacing Actinobacteria as the fourth most abundant phylum (Figure 2; Supplementary Figure 6; Table 3). Similar to the healthy subject group, Firmicutes were the most abundant phyla. When stratified according to sample type, there was a notable overabundance of Fusobacteria in CRC samples (8.6% in tumour tissue compared with 0.8% in adjacent normal mucosa *p* = 0.009, Wilcoxon rank-sum test; Table 3), an observation which confirms previous findings (39–42). Interestingly, the increased abundance in CRC samples was only present in the samples of overweight patient (*p* = 0.029, Wilcoxon rank sum test) and not in healthy weight individuals (*p* > 0.05, Wilcoxon rank sum test). Fusobacteria presence in the samples of the healthy controls was determined, with a relative abundance of less than the normal mucosa of CRC patient (0.25% compared with 0.8%). When stratified according to BMI, Fusobacteria abundance was higher in the overweight healthy control group (0.46%) compared with the normal weight group (0.004%), although this was not statistically significant.

**Table 3:** Mean difference in the relative abundance of the phyla Firmicutes, Bacteroidetes, Proteobacteria, and Actinobacteria in healthy subjects stratified according to BMI status. Healthy weight (BMI of 20–25 kg/m^2^), overweight (BMI of > 25 kg/m^2^).

**Phylum**	**Mean relative abundance** **±SEM**		**Difference in mean relative abundance ±SEM**	***p*-value**
	**Healthy weight**	**Overweight**		
Firmicutes	51.04 ± 5.49	48.79 ± 8.89	2.24 ± 3.40	0.429
Bacteroidetes	45.14 ± 5.53	46.37 ± 8.88	1.23 ± 3.35	0.537
Proteobacteria	2.69 ± 0.79	3.10 ± 0.74	0.41 ± 0.05	0.792
Actinobacteria	0.42 ± 0.16	0.49 ± 0.23	0.07 ± 0.01	0.792

*The value of p based on Wilcoxon rank-sum test (to 3 d.p.). Healthy weight (BMI of 20–25 kg/m^2^), overweight (BMI of > 25 kg/m^2^)*.

**Table 4:** Mean difference in the relative abundance of the 4 major phyla Firmicutes, Bacteroidetes, Proteobacteria, and Fusobacteria from the CRC sample cohort between polyp and paired normal samples, and between normal weight and overweight patient samples.

**Phylum**	**Mean relative abundance between CRC and Adjacent normal mucosa** **±SEM**		**Difference in mean relative abundance ±SEM**	***p*-value**	**Mean relative abundance between Healthy and High BMI groups** **±SEM**		**Difference in mean relative abundance ±SEM**	***p*-value**
	**Tumour**	**Adjacent normal mucosa**			**Healthy weight**	**Overweight**		
Firmicutes	51.19 ± 3.96	51.12 ± 3.60	0.07 ± 0.36	0.862	54.11 ± 3.50	48.20 ± 3.94	5.91 ± 0.44	0.201
Bacteroidetes	32.96 ± 3.07	38.56 ± 3.12	5.59 ± 0.05	0.165	31.17 ± 2.68	40.35 ± 3.26	9.18 ± 0.58	**0.049**
Proteobacteria	5.36 ± 0.80	5.61 ± 1.20	0.25 ± 0.40	0.659	6.46 ± 1.22	4.51 ± 0.69	1.95 ± 0.53	0.265
Fusobacteria	8.57 ± 3.21	0.79 ± 0.41	7.78 ± 2.80	**0.009**	6.31 ± 3.21	3.05 ± 1.23	3.26 ± 1.98	0.675

*The value of p based on Wilcoxon rank-sum test (to 3 d.p.)*.

**Figure 3:**
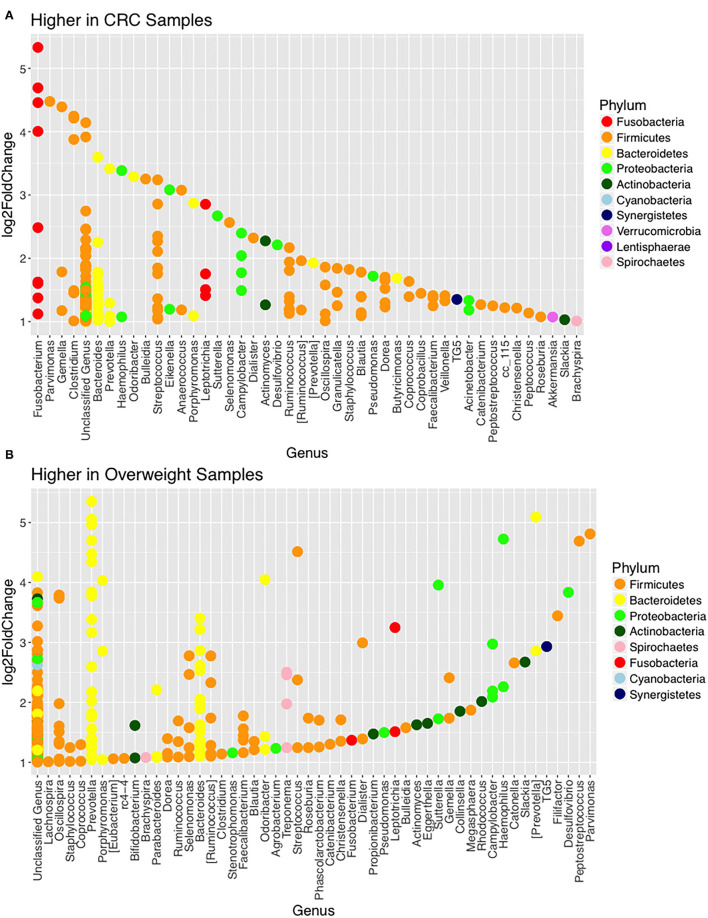
Changes in relative abundance of operational taxonomic units (OTUs). **(A)** OTUs with LogFC > 1 with higher abundance in CRC samples (when compared with adjacent normal samples) including *Fusobacterium* (adj *p* = 0.014; DESeq2) and **(B)** OTUs with LogFC > 1 with higher abundance in overweight samples (when compared with healthy weight), in the CRC patient cohort, including *Prevotella copri* (adj *p* = 0.042; DESeq2).

Further interrogation of the CRC patient cohort, based on BMI status, showed that overweight individuals had higher levels of Bacteroidetes than their lean counterparts (31.17% in the samples of normal weight patient, 40.35% in the samples of overweight patient; *p* = 0.049; Table 3). In particular, an increase in *Prevotella* was observed in patients within the higher BMI group (Supplementary Figure 7). To further interrogate the influence of BMI and sample type in the samples of CRC patients, differential abundance analysis was conducted using DESeq2. This methodology has been shown to reduce false positive rates in discovery of significant abundance differences when compared with typical rarefaction methods (43). DESeq2 analyses further supported these taxonomic differences with *Fusobacterium* identified as differing between adjacent normal mucosa and CRC samples (adj *p* < 0.005, Figure 3A; Supplementary Table 2), and *Prevotella copri* seen to have significant differences in abundance between healthy weight and overweight patient samples (adj *p* < 0.05, Figure 3B; Supplementary Table 2).

**Figure 4:**
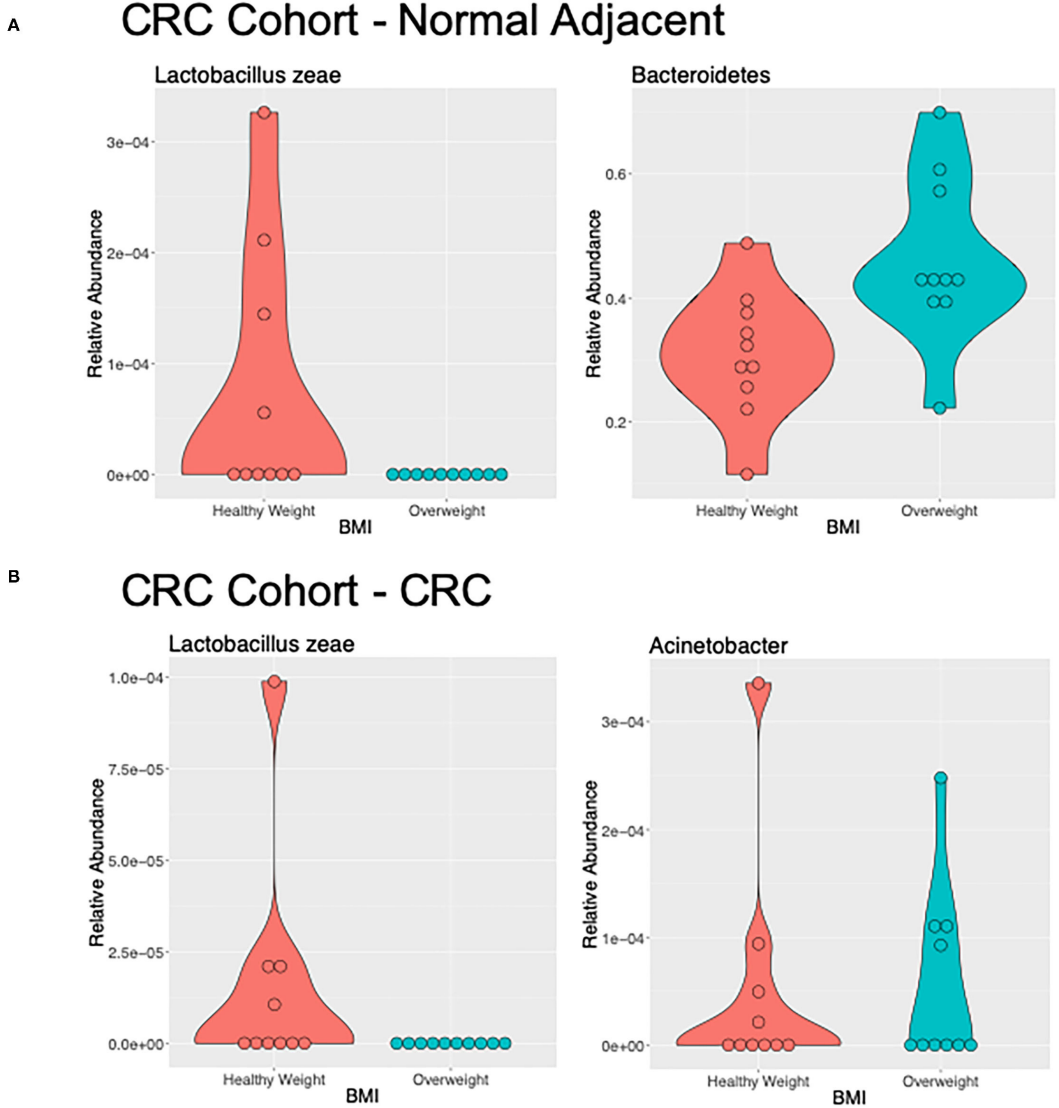
Differentially abundant genera of interest between the samples of healthy weight and overweight patient from **(A)** adjacent normal mucosa samples from patients with CRC, and **(B)** CRC samples by LefSe (LDA > 2).

We used LEfSe to identify OTUs that were driving the differences between BMI stratified samples in the two subject groups. There were no significant differences between healthy weight and overweight subject samples in the healthy subject cohort. Discriminant feature analysis of the CRC cohort showed *Bacteroides* and Tissierellaceae were over-represented in adjacent normal mucosa in overweight patients, compared with adjacent normal mucosa in healthy weight patients, and *Lactobacillus zeae*, was over-represented in adjacent normal mucosa in the healthy weight patients with CRC (Figure 4A; Supplementary Table 3). *Acinetobacter* was increased in CRC samples from the overweight group. Similar to the adjacent normal mucosa samples, *Lactobacillaceae zeae* was seen in higher abundance in healthy weight CRC patient samples (Figure 4B; Supplementary Table 3).

We further interrogated the dataset to allow us to identify which specific cohort parameters were influencing the observed microbial diversity changes. We used Bray Curtis and Jaccard distance measures, which revealed that samples did not cluster strongly by BMI status in the healthy subject cohort (*p* > 0.05; PERMANOVA; Table 4; Supplementary Figure 8). However, interrogation of the CRC cohort suggested that BMI status significantly contributed to the distance between samples (*p* < 0.05; PERMANOVA; Table 4; Supplementary Figure 9).

**Table 5:** PERMANOVA results produced by Adonis of the R package Vegan showing the contribution of each metadata category to the sample distances.

**Metadata category**	**p-value for Bray Curtis distance matrix**	***p*-value for Binary Jaccard distance matrix**
**Healthy sample cohort**		
BMI	0.258	0.147
**CRC cohort**		
Sample type	0.437	0.996
BMI	0.025	0.009
Patient	0.069	0.015

### Microbiome Interaction Networks Are Influenced by BMI Status as Well as Disease State

**Figure 5:**
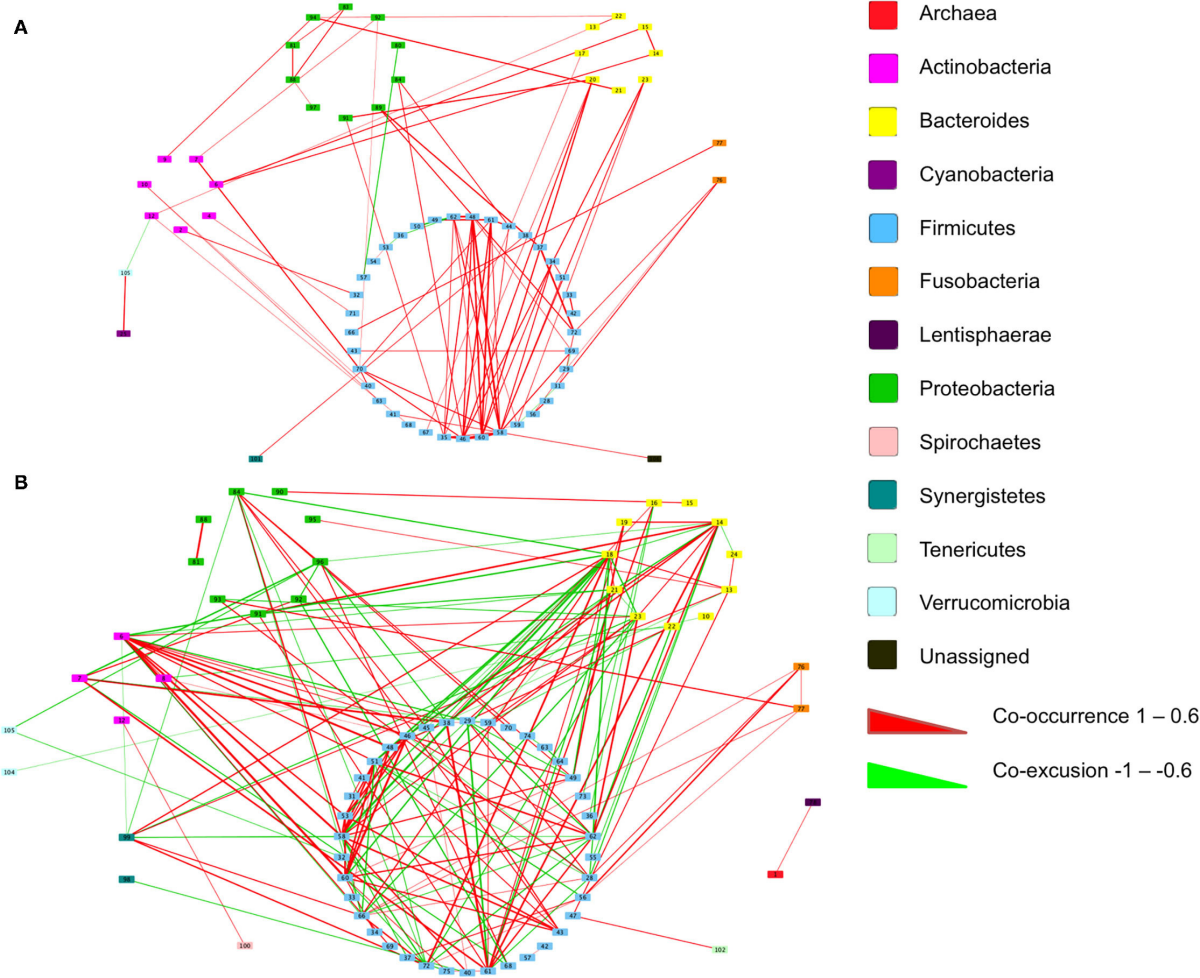
Co-occurrence networks of taxa grouped by phyla in the samples of patient with CRC. Relationships with a correlation > 0.6 or < −0.6 and an adjusted *p* < 0.05 are presented from **(A)** normal BMI samples and **(B)** high BMI samples. Co-occurrence (red) and co-exclusion (green) relationships are represented by weighted edges, based on the strength of the correlation. Taxa corresponding to each identification number are available in Additional File 1.

We next inferred all pairwise taxonomic correlations between adjacent normal mucosa and CRC samples, with BMI status as a classifier, using the SparCC algorithm. After correcting for spurious correlation coefficients and controlling for false discovery rates, we were able to see that BMI status impacted on the number of observed taxonomic correlations (Figure 5; Additional File 1). We found the highest number of significant positive correlations in the samples of overweight patient with CRC (BH adjusted *p* < 0.05; Figure 5). Samples from healthy weight patients with CRC had a total of 88 significant correlations (81 co-occurrence and 7 co-exclusion) across 70 taxa. When additionally stratified by sample type (adjacent normal mucosa or CRC), adjacent normal mucosa samples had 41 significant correlations compared with only 15 in the CRC samples. When a similar assessment was undertaken on the samples from the high BMI CRC patient group, the number of significant correlations increased dramatically to 184 (108 co-occurrence and 76 co-exclusion), although the number of taxa did not increase indicating the increase observed reflected an increase in networking within a similarly rich community. Additional stratification based on the sample type showed that adjacent normal mucosa samples had 25 correlations compared with 29 in CRC samples. Trans-phylum relationships, with strong correlation, (0.6 or above) were much more common in the higher BMI group compared with the samples of normal weight patient with CRC, however, no difference was seen based on the sample type indicating that the co-occurrence networks were driven by BMI status rather than the presence of CRC. Similar to findings from Nakatsu et al. (19), Firmicutes members were more likely to form strong co-occurring relationships indicating that specific gut microbiota members can form niche-specific relationships, which in our study appear to be a response to the increased BMI status. Network analysis identified very little overlap in the co-occurrence networks between healthy weight and overweight patient sample sets indicating that the increases seen reflected a progressive alteration from healthy weight to overweight patient samples. The strongest interactions were among various Firmicutes belonging to *Bulledia, Dorea* and *Ruminococcus* co-occurring with Bacteroidetes members, such as *Prevotella* and *Rikenella*, although there was evidence of a co-exclusion relationship between *Ruminococcus* and *Prevotella* (Additional File 1). Other associations included uncultured Oscillospira forming strong co-occurrence relationships with *Coriobacteria* as well as *Barnsiella*. Interestingly, a number of Firmicutes, such as *Faecalibacterium, Ruminococcus*, and *Blautia* were shown to co-occur with *Bifidobacterium*. The strongest co-exclusion networks were seen between *Selenomonas* and other Firmicutes, such as *Lachnospiraceae* and *Ruminococcaceae* but also Bacteroidetes and Actinobacteria. These were only seen in overweight patients.

## Discussion

It is expected that CRC burden will substantially increase in the next two decades as a consequence of adoption of a western lifestyle (1). However, to date there is limited information related to whether BMI status influences the microbial composition in the context of CRC. In this study, we investigated how a BMI of >25 kg/m^2^ influenced mucosal associated microbial communities in patients with CRC. We compared the findings to control subjects of equivalent BMI status, from the same demographic area but with no colonic pathology. The findings demonstrate that BMI status influences the microbial community structure in patients with CRC. In particular, we show that in patients with CRC, an increased BMI was associated with more dynamic microbial networks evidenced by the increased numbers of co-occurring and co-exclusion relationships between microbes which may highlight a BMI-directed colonic tumour environment. Previous studies have demonstrated that differences in gut microbial communities are present through the various stages of the adenoma-carcinoma sequence (19, 44, 45). These changes have been proposed to happen, at least in part, in response to the changing colonic environment as carcinogenesis progresses, such as increased inflammatory activity, altered host energy metabolism, and increased cell turnover (46–48). In addition, our findings show that BMI status contributes to the mucosa-associated microbial community shifts, in particular having a BMI of >25 kg/m^2^ was associated with an overabundance of *Prevotella* in patients with CRC. *Prevotella* sp. have been repeatedly demonstrated to be associated with obesity induced disease (49–51) as well as being under-represented in non-obese subjects (52), and being detected in CRC microbial signatures (53). Recently *Prevotella copri* was shown to be associated with fat accumulation in pigs (54). Whilst the majority of information linking *Prevotella* abundance with weight gain has been in the context of obesity, our study has extended the findings to a CRC cohort with BMI of >25 kg/m^2^. This means that increased *Prevotella* abundance is present prior to obesity. *Prevotella* are known to play a role in carbohydrate fermentation, producing exogenous short-chain fatty acids, such as succinate, as well as producing sialidases which degrade mucin affecting the mucosal barrier integrity (54–57). It has been shown that hydrogen-producing *Prevotella* can coexist with hydrogen-oxidising methanogenic Archaea in the gastrointestinal tracts of individuals with a high BMI (55). This syntrophic relationship may increase the host energy extraction from indigestible carbohydrates, as an increase in hydrogen-oxidising methanogenesis facilitates fermentation. Therefore, Prevotellaceae populations may be an important factor in the association among increased BMI status, CRC, and the gut microbiota. It remains to be determined how such changes in the gut microbiota, and the accompanying impact on microbial function affect the host during CRC development. Future studies focussing on defining the tumour-promoting potential of *Prevotella* are warranted to assess how individual species interact and contribute to the tumourigenic process. This is particularly pertinent as there have been conflicting reports of the beneficial as well as deleterious effects of *Prevotella* species, depending on the nature of the environment (58, 59).

Additionally, our findings demonstrate an overabundance of Fusobacteria in CRC samples, with CRC tissue harbouring a higher Fusobacteria load compared with adjacent normal mucosa which confirms previous findings (39, 60, 61) with a lower Fusobacteria abundance seen in the mucosa of healthy subject. Comparison of Fusobacteria abundance in normal mucosa between patients with CRC and healthy subjects in the same geographical cohort is scarce. Our findings agree with a previous quantitative PCR study which compared Fusobacteria abundance in the mucosal samples of patients with CRC and healthy subjects (62). Our findings of increased BMI, independent of disease status, also correlating with increased Fusobacteria, is novel and worthy of further exploration. Although Fusobacteria abundance in healthy subject levels was lower than the levels of patients with CRC, the same trend of increased Fusobacteria abundance correlating with increased BMI was seen between both groups. To our knowledge, this is the first report of an association specifically with overweight patients with CRC. Fusobacteria, particularly *Fusobacterium nucleatum*, along with enterotoxigenic *Bacteroides fragilis* and *Escherichia coli* have been described as putative bacterial oncogenic drivers of CRC and their role in colorectal tumorigenesis has been repeatedly demonstrated, with the presence of *F. nucleatum* especially reported to contribute to disease progression, chemoresistance, and metastatic disease (53, 63, 64). Recent attention has focussed on the oncogenic potential of collective gut microbial communities, such as the role of bacterial biofilms, rather than individual contributors, with oncogenic driver organisms known to be the key constituents of these polymicrobial biofilms (65–67). Within biofilms, microorganisms become resistant not only to host defence mechanisms, but also to anti-microbial strategies with invasive polymicrobial bacterial biofilms being a known driver of tissue inflammation. It has been previously demonstrated that *F. nucleatum* plays a central role in oral biofilm formation, mediating coaggregation between strains including various *Prevotella* species (68). Whether *F. nucleatum* plays such a pivotal role in gut biofilms remains to be determined, however, a recent study has shown that *Fusobacterium* and its associated microbiome—such as *Bacteroides, Selenomonas*, and *Prevotella* species, present in CRC primary lesions are also present in distal metastases suggesting that *Fusobacterium* has some ability to direct its environment (53).

A strength of our experimental design was the inclusion of paired samples, with histologically normal mucosa, from near the CRC site of the lesion. By using paired samples, each individual acted as their own control, providing a higher level of comparability. Selected previous studies, have also opted to use this approach (19, 39, 61), although most other studies either use healthy individuals as controls or more often relying on the faecal sample comparison between individuals. The paired patient sample approach provides the best benchmark of microbial diversity for each individual as it is widely appreciated that there is no “gold standard” definition of the microbial composition of the healthy or normal gut microbiota. Limitations of our study include small sample size and also the inevitable effect of bowel cleansing preparation on the mucosa-associated microbiota. However, this is a caveat of all studies which look to obtain colonic samples and the assumption would be that all subjects were affected to a similar extent as they have undergone almost identical procedures. A further limitation of the study was the fact that no anthropomorphic assessment of patients was undertaken to define whether the increased BMI status was due to increased body fat or muscle mass. Previous studies assessing gut microbial communities in athletes with an increased BMI compared with individuals with an increased BMI due to carrying excess body fat, have shown that gut microbial communities differ dramatically depending on the body composition (69, 70). Based on the age range of our cohort, which was adults with an average age of >60 years old, we anticipate that the increased BMI cohort was reflective of the population demographic from which they were recruited.

## Conclusion

In summary, our study has shown that carrying excess body weight influences mucosal microbial community structure in patients with CRC. We anticipate that evaluating the mucosal microbial community composition and progression alongside host responses will provide a clearer picture of how carrying excess bodyweight influences the CRC development. Although further confirmation of our findings is needed, studies are warranted to define the mechanistic link between *Prevotella* overabundance and increased BMI status in the context of CRC. This information may enable earlier screening to predict patients at risk of developing CRC and allow prevention strategies to be implemented.

## Data Availability Statement

The datasets presented in this study can be found in online repositories. The names of the repository/repositories and accession number(s) can be found below: https://www.ebi.ac.uk/ena, PRJEB15003, and PRJEB22039.

## Ethics Statement

The studies involving human participants were reviewed and approved by the North of Scotland Research Ethics Service (Study Codes 09/S0802/106, 12/NS/0061). The patients/participants provided their written informed consent to participate in this study.

## Author Contributions

GH conceived the study. SS and SB performed all the analyses. JT collected clinical samples. EE-O collected clinical samples and provided critical evaluation of the manuscript. GM assessed colonic pathology of biopsy and surgical resection samples. GH and GM secured funding for the study through a grant awarded by Friends of Anchor. SS and GH interpreted the data and wrote the manuscript. All authors read and approved the final manuscript.

## Conflict of Interest

The authors declare that the research was conducted in the absence of any commercial or financial relationships that could be construed as a potential conflict of interest.

## Publisher's Note

All claims expressed in this article are solely those of the authors and do not necessarily represent those of their affiliated organizations, or those of the publisher, the editors and the reviewers. Any product that may be evaluated in this article, or claim that may be made by its manufacturer, is not guaranteed or endorsed by the publisher.
