# Supplementary material for: Gut Mucosal Microbiome Signatures of Colorectal Cancer Differ According to BMI Status
Source: Front Med (Lausanne). 2022 Feb 8;8:800566. doi: 10.3389/fmed.2021.800566 (PMC8861504; doi:10.3389/fmed.2021.800566)
Supplement: Supplementary file 1 [file Data_Sheet_1.docx]

**Supplementary Figure 1: Rarefaction Analysis for all sample cohorts**. Rarefaction curves for (A) observed species measure from CRC cohort samples, (B) Chao diversity measure from CRC cohort samples, (C) observed species measure from healthy patient samples, and (D) Chao diversity measure from healthy patient samples.


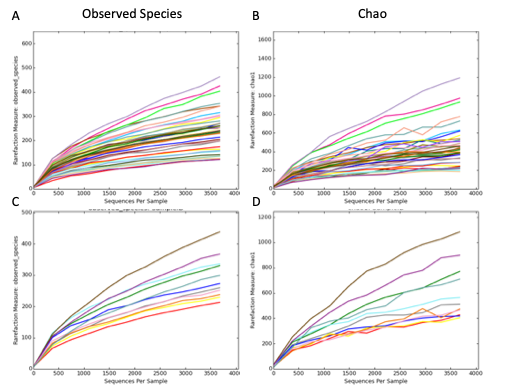


**Supplementary Figure 2: Species diversity comparison between healthy subjects normal colon samples stratified by BMI status.** Microbiota structure and composition were measured using Observed OTUs and Chao (species richness), Shannon-Weiner diversity index, Inverse Simpson’s evenness index and Good’s coverage (species richness). Alpha diversity scores calculated by subsampling samples to 3689 reads. Each point represents the diversity score for a patient sample. Error bars represent SEM. Between-group variations were measured using Wilcoxon Rank test.

**
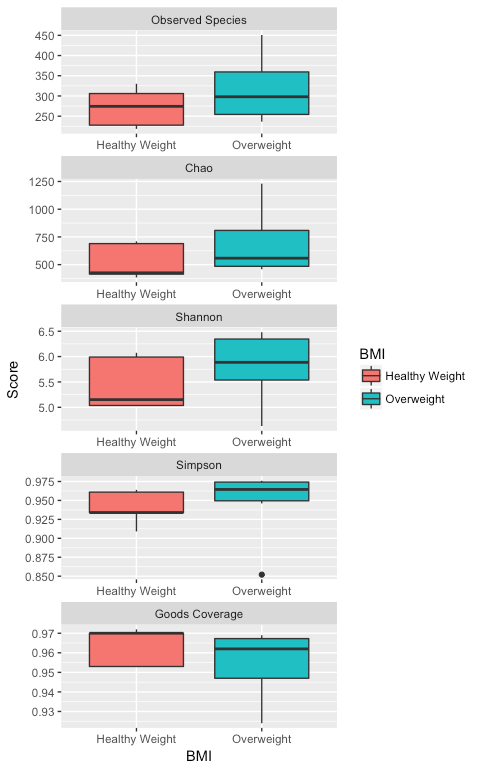
**

**Supplementary Figure 3: Species diversity comparison between (A) paired colorectal cancer/adjacent normal mucosa samples (B) all samples stratified by BMI status and (C) paired colorectal cancer/adjacent normal mucosa samples stratified by BMI status.** Microbiota structure and composition were measured using Observed OTUs and Chao (species richness), Shannon-Weiner diversity index, Inverse Simpson’s evenness index and Good’s coverage (species richness). Alpha diversity scores calculated by subsampling samples to 3689 reads. Each point represents the diversity score for a patient sample. Error bars represent SEM. Between-group variations were measured using Wilcoxon Rank test.


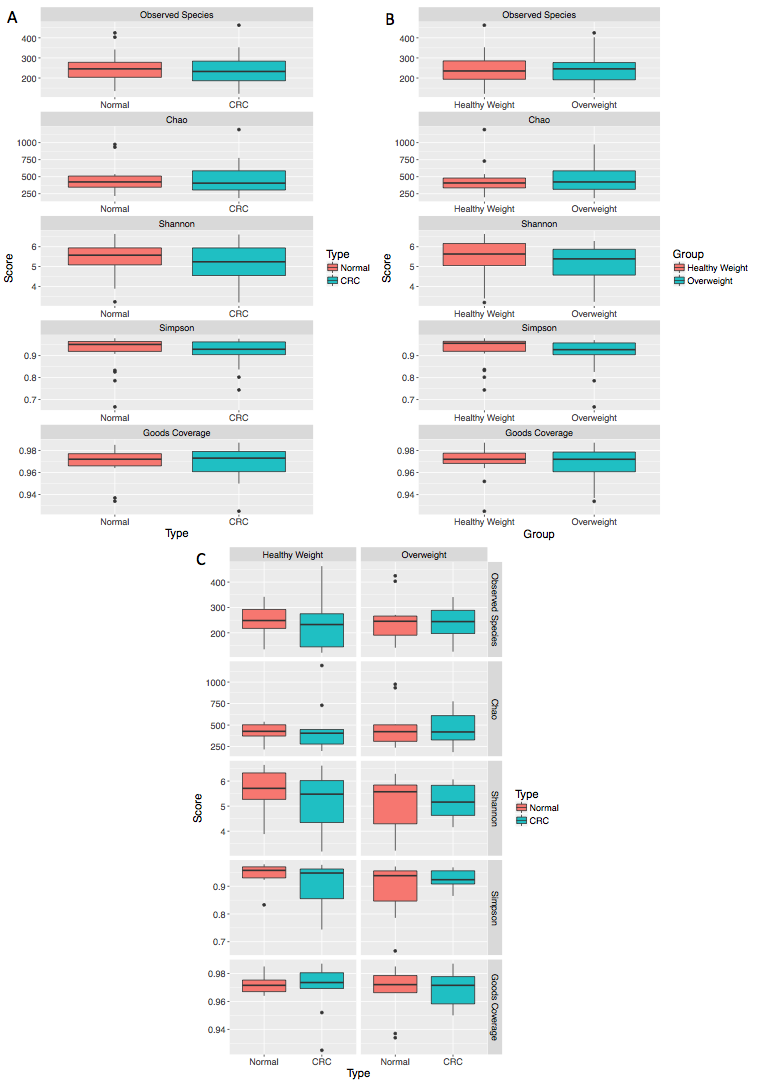


**Supplementary Figure 4: Species diversity comparison between healthy participant normal colon samples and normal mucosa adjacent samples from CRC patients stratified by A) sample type and B) BMI and sample type.** Microbiota structure and composition were measured using Observed OTUs and Chao (species richness), Shannon-Weiner diversity index, Inverse Simpson’s evenness index and Good’s coverage (species richness). Alpha diversity scores calculated by subsampling samples to 3689 reads. Each point represents the diversity score for a patient sample. Error bars represent SEM. Between-group variations were measured using Wilcoxon Rank test.

**
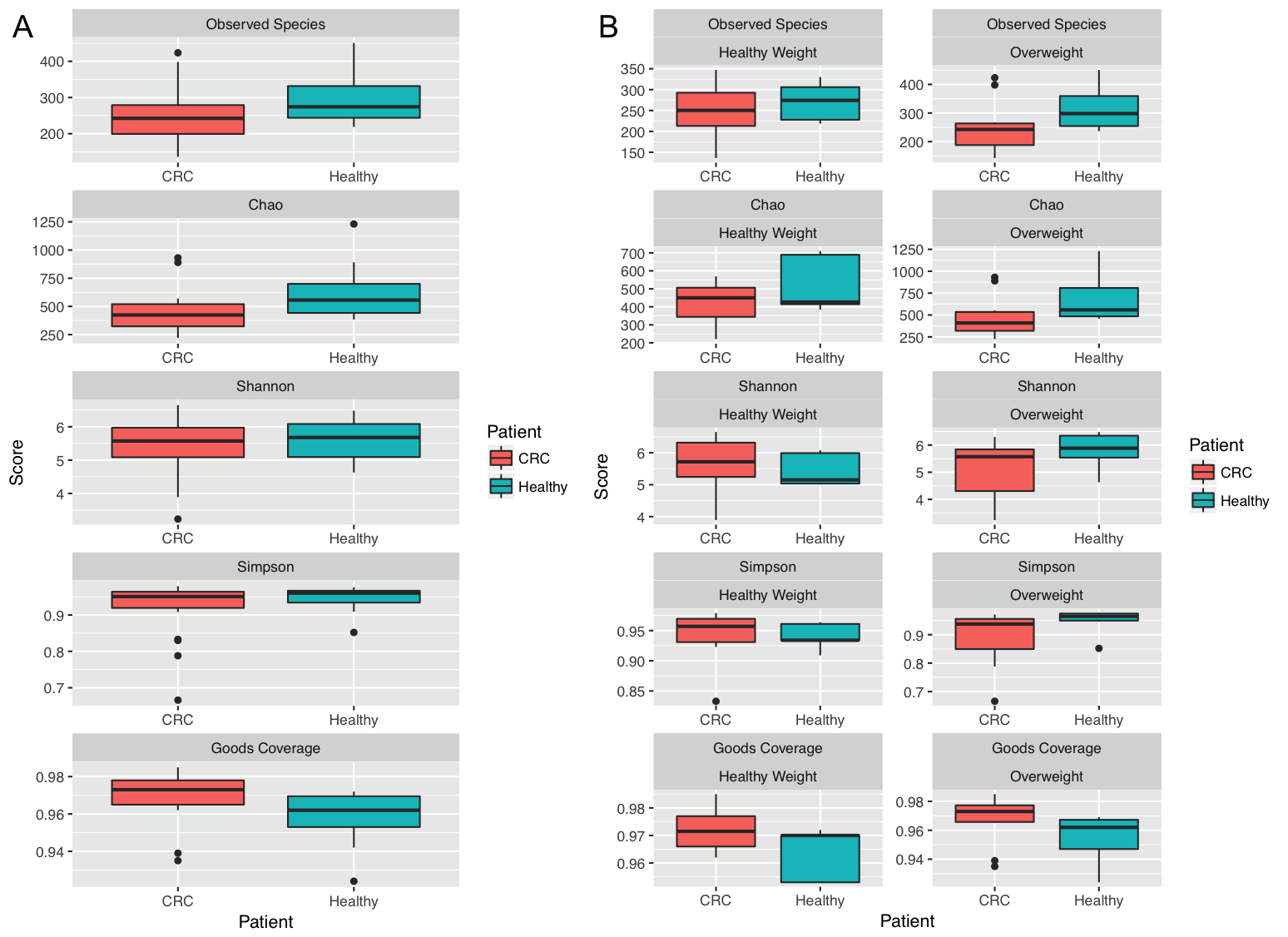
**

**Supplementary Figure 5: The distribution of bacteria in healthy subjects at (A) Phylum level and (B) Family level.** Heatmaps show Log2count of sequences within each classification level. Colours on the column caps: Green/purple depicts BMI status (Purple = healthy weight (20-25kg/m^2^), Green = overweight (above 25kg/m^2^).


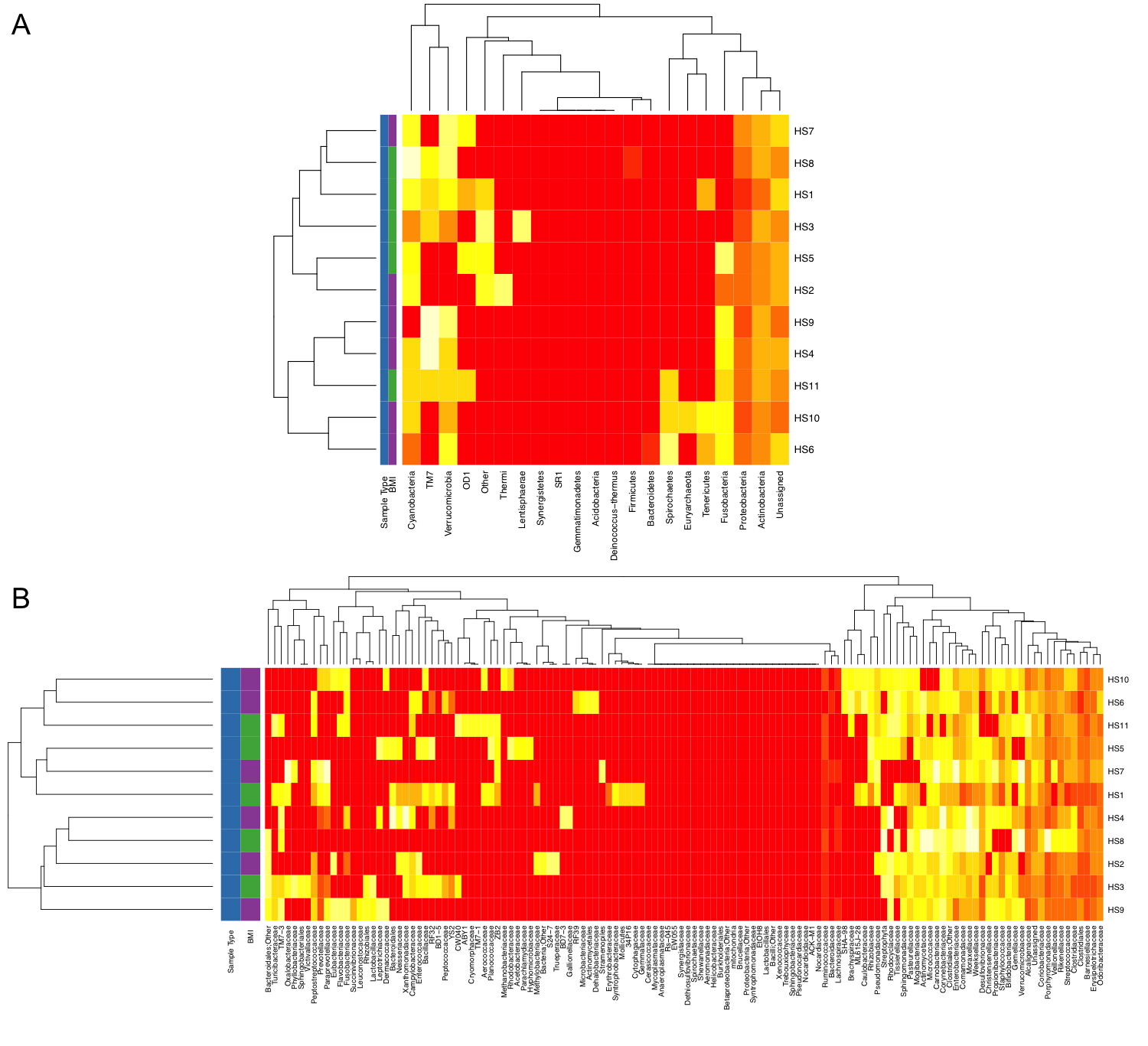


**Supplementary Figure 6: The distribution of bacteria in colorectal cancer samples and paired normal samples at (A) Phylum level and (B) Family level.** Heatmaps show Log2count of sequences within each classification level. Two sets of colours on the column caps: Red/Blue depicts sample type (Red = normal (N), Blue = CRC (C)), Green/purple depicts BMI status (Purple = healthy weight (20-25kg/m^2^), Green = overweight (above 25kg/m^2^).


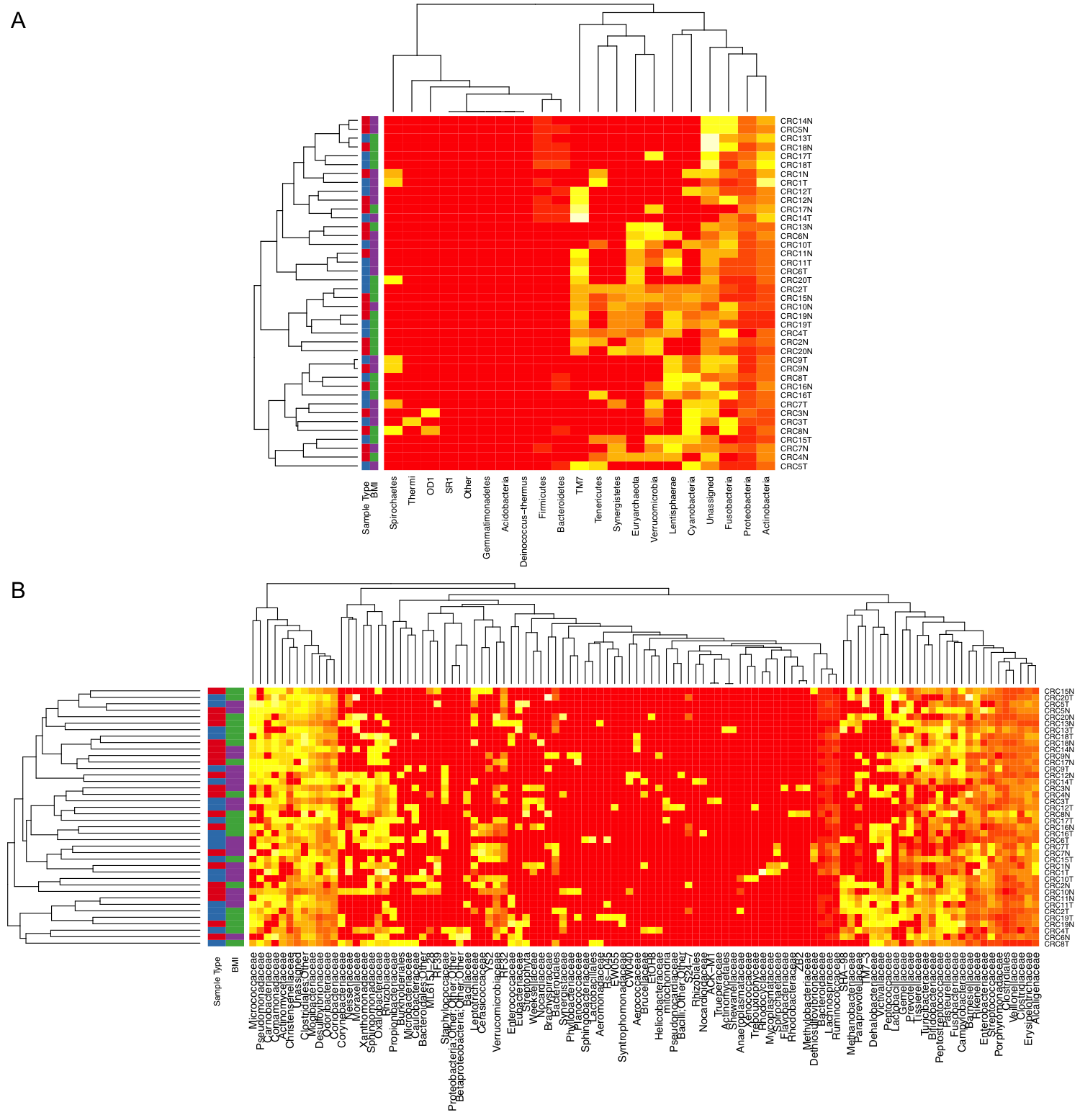


**Supplementary Figure 7. Relative abundance at genus level for CRC and paired normal samples highlighting the increased abundance of *Prevotella* (depicted in red) in patients with a BMI > 25kg/m^2^.**

**
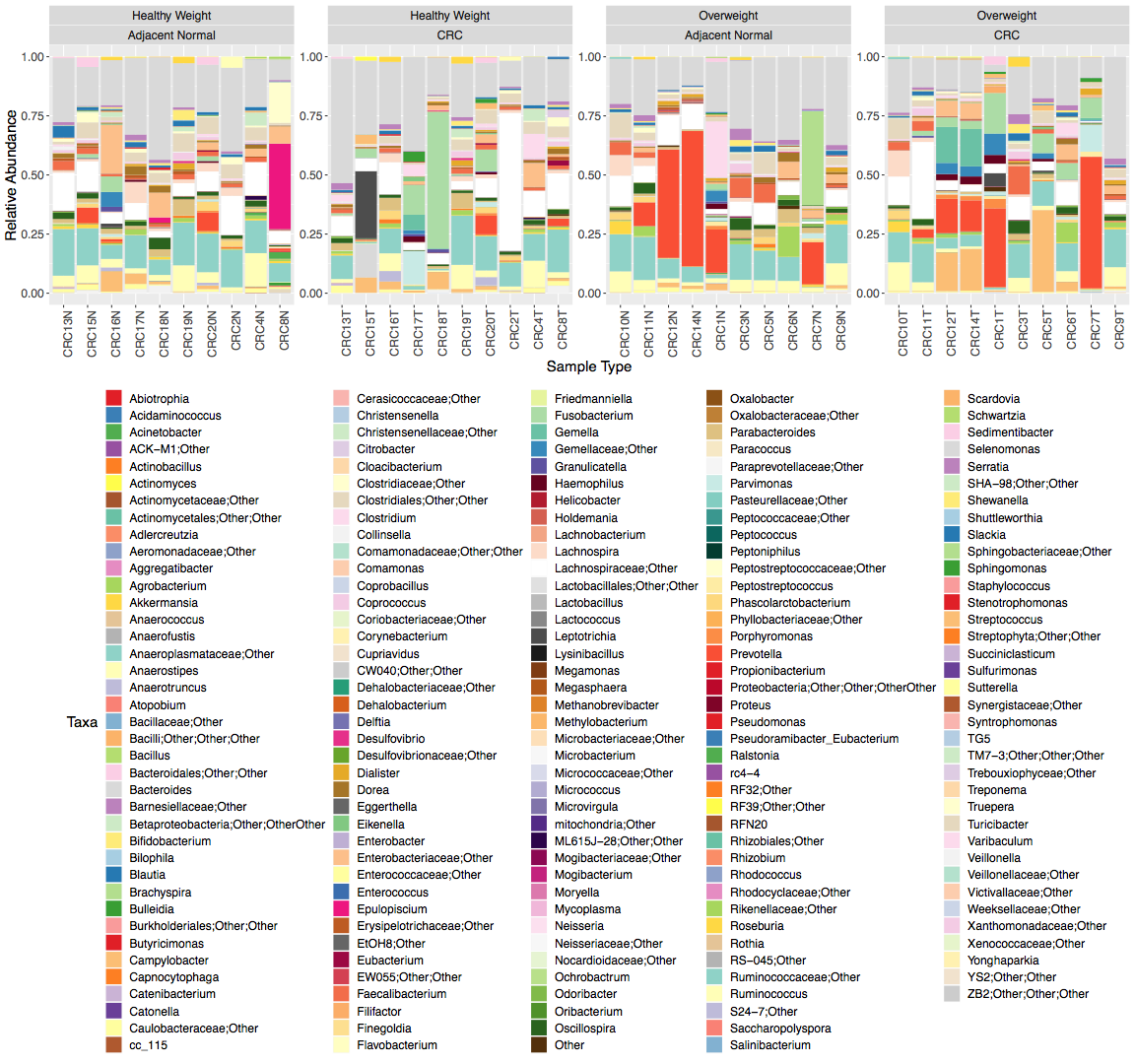
**

**Supplementary Figure 8: Beta diversity comparisons for healthy patient cohort**. Clustering of samples according to patient BMI (Blue = 20 - 25kg/m^2^, Red = above 25kg/m^2^) by PCoA, based on Bray-Curtis similarity distance. Within the PCoA plots loadings of the 3 axes sum up to 50.29%.


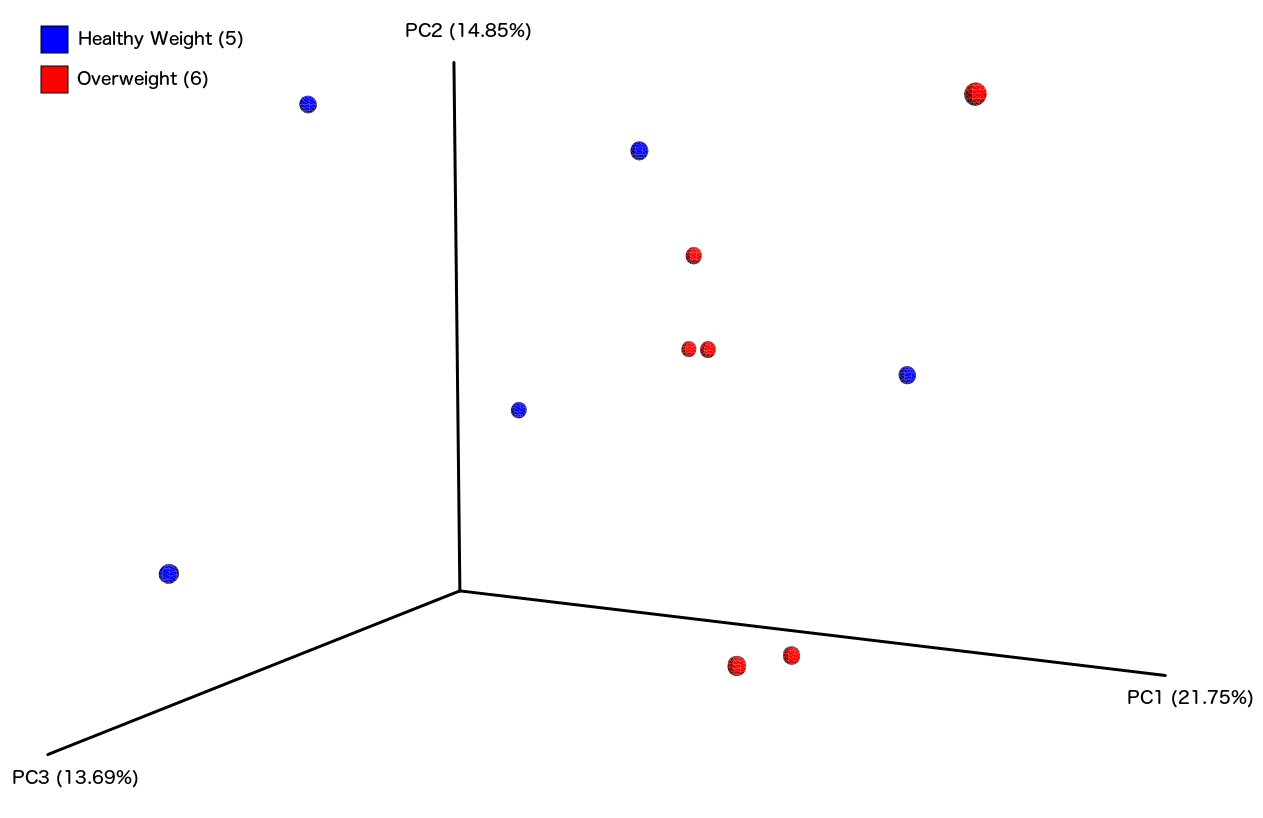


**Supplementary Figure 9: Beta diversity comparisons for CRC cohort**. Clustering of samples according to (A) and (B) sample type (CRC and paired normal mucosa) and (C) and (D) patient BMI (Blue = 20 - 25kg/m^2^, Red = above 25kg/m^2^) by PCoA, based on Bray-Curtis similarity distance. Within the PCoA plots loadings of the 3 axes sum up to 32.04%. (B) and (D): CRC and paired normal mucosa samples from the same individual are connected together. Each subject is depicted in a unique colour.


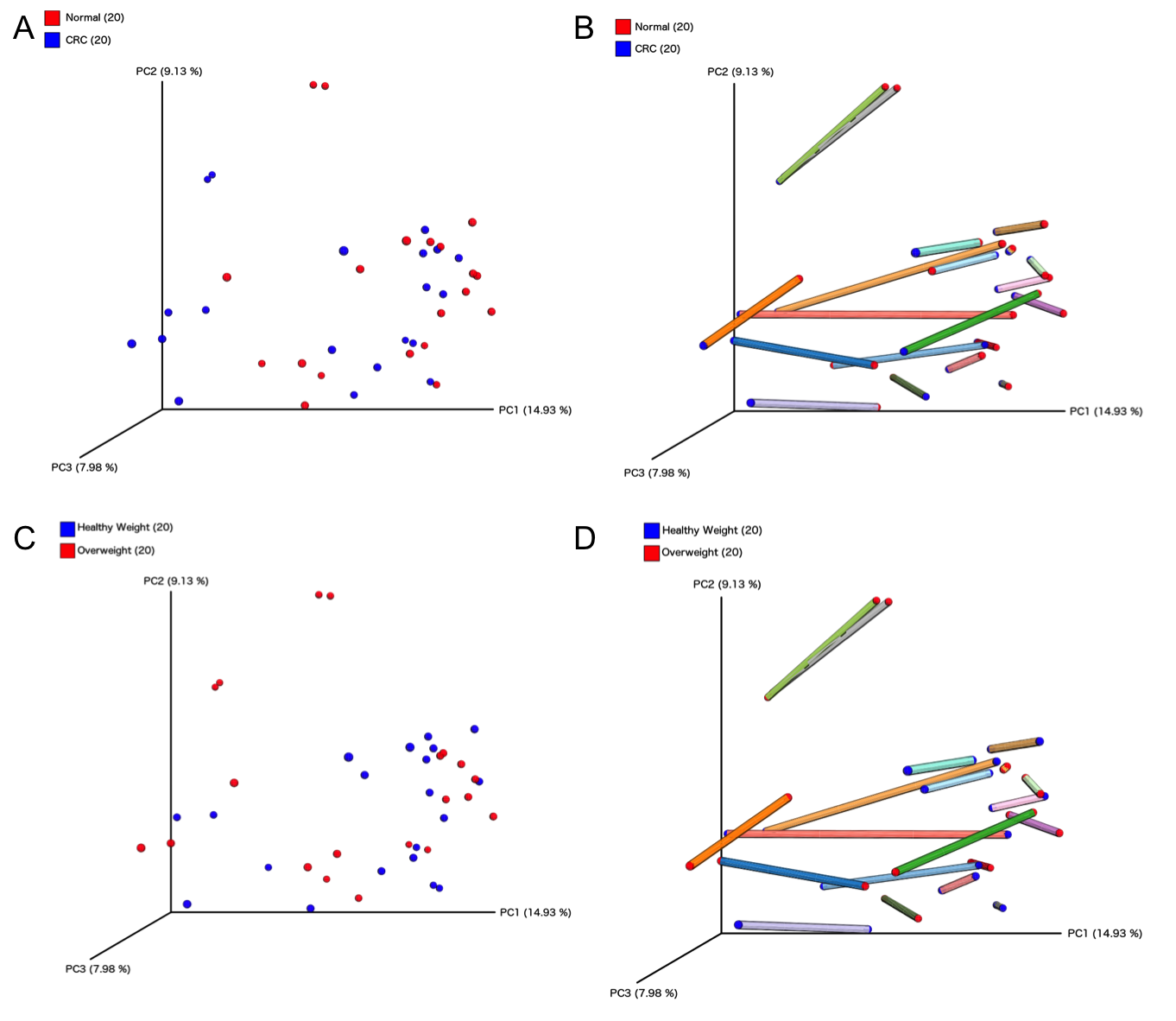


**Supplementary Table 1A - CRC cohort read statistics**

| **SampleID** | **Sequencing File Prefix** | **Sample Type** | **BMI Status** | **# of Raw Reads** | **# of Filtered Reads** |
| --- | --- | --- | --- | --- | --- |
| CRC1N | TBID5291-N | Normal | Overweight | 36696 | 21202 |
| CRC1T | TBID5291-T | Tumour | Overweight | 132247 | 97363 |
| CRC2N | TBID5294-N | Normal | Healthy Weight | 54920 | 43771 |
| CRC2T | TBID5294-T | Tumour | Healthy Weight | 46277 | 30588 |
| CRC3N | TBID5295-N | Normal | Overweight | 48280 | 24356 |
| CRC3T | TBID5295-T | Tumour | Overweight | 74387 | 47965 |
| CRC4N | TBID5314-N | Normal | Healthy Weight | 39918 | 18552 |
| CRC4T | TBID5314-T | Tumour | Healthy Weight | 94586 | 66232 |
| CRC5N | TBID5331-N | Normal | Overweight | 41139 | 30805 |
| CRC5T | TBID5331-T | Tumour | Overweight | 99177 | 71362 |
| CRC6N | TBID5332-N | Normal | Overweight | 133575 | 103108 |
| CRC6T | TBID5332-T | Tumour | Overweight | 98023 | 65392 |
| CRC7N | TBID5354-N | Normal | Overweight | 58249 | 45525 |
| CRC7T | TBID5354-T | Tumour | Overweight | 67134 | 46834 |
| CRC8N | TBID5355-N | Normal | Healthy Weight | 23170 | 9056 |
| CRC8T | TBID5355-T | Tumour | Healthy Weight | 131282 | 96291 |
| CRC9N | TBID5357-N | Normal | Overweight | 29676 | 17128 |
| CRC9T | TBID5357-T | Tumour | Overweight | 47948 | 33690 |
| CRC10N | TBID5364-N | Normal | Overweight | 106311 | 76979 |
| CRC10T | TBID5364-T | Tumour | Overweight | 61099 | 32564 |
| CRC11N | TBID5365-N | Normal | Overweight | 53350 | 37771 |
| CRC11T | TBID5365-T | Tumour | Overweight | 48876 | 32363 |
| CRC12N | TBID5378-N | Normal | Overweight | 111714 | 78371 |
| CRC12T | TBID5378-T | Tumour | Overweight | 49594 | 23218 |
| CRC13N | TBID5381-N | Normal | Healthy Weight | 68151 | 50222 |
| CRC13T | TBID5381-T | Tumour | Healthy Weight | 33441 | 21762 |
| CRC14N | TBID5398-N | Normal | Overweight | 90983 | 66158 |
| CRC14T | TBID5398-T | Tumour | Overweight | 29407 | 12115 |
| CRC15N | TBID5399-N | Normal | Healthy Weight | 54236 | 39139 |
| CRC15T | TBID5399-T | Tumour | Healthy Weight | 82724 | 57389 |
| CRC16N | TBID5405-N | Normal | Healthy Weight | 48958 | 27306 |
| CRC16T | TBID5405-T | Tumour | Healthy Weight | 64367 | 44318 |
| CRC17N | TBID5420-N | Normal | Healthy Weight | 32126 | 16947 |
| CRC17T | TBID5420-T | Tumour | Healthy Weight | 94487 | 72003 |
| CRC18N | TBID5450-N | Normal | Healthy Weight | 57028 | 36624 |
| CRC18T | TBID5450-T | Tumour | Healthy Weight | 70353 | 49227 |
| CRC19N | TBID5452-N | Normal | Healthy Weight | 94315 | 71830 |
| CRC19T | TBID5452-T | Tumour | Healthy Weight | 82311 | 62476 |
| CRC20N | TBID5457-N | Normal | Healthy Weight | 95589 | 72743 |
| CRC20T | TBID5457-T | Tumour | Healthy Weight | 65490 | 47572 |

**Supplementary Table 1B - Healthy subject cohort read statistics**

| **Sample** | **Sequencing File Prefix** | **BMI Status** | **# of Raw Reads** | **# of Filtered Reads** |
| --- | --- | --- | --- | --- |
| HS1 | TestBx10 | Healthy Weight | 62229 | 23019 |
| HS2 | TestBx11 | Overweight | 88604 | 34114 |
| HS3 | TestBx13 | Healthy Weight | 121464 | 67988 |
| HS4 | TestBx17 | Overweight | 127637 | 76820 |
| HS5 | TestBx20 | Healthy Weight | 71225 | 23507 |
| HS6 | TestBx21 | Overweight | 67693 | 31338 |
| HS7 | TestBx22 | Overweight | 76871 | 25681 |
| HS8 | TestBx24 | Healthy Weight | 99180 | 59545 |
| HS9 | TestBx27 | Overweight | 131330 | 65520 |
| HS10 | TestBx32 | Overweight | 94501 | 28209 |
| HS11 | TestBx4 | Healthy Weight | 78435 | 30964 |

**Supplementary Table 2. Sequence variants with a significant change in abundance as identified by DESeq2 between all adjacent normal and CRC tumour samples, and between all healthy weight and overweight samples, from the CRC patient cohort.** Significance determined as adjusted p value < 0.05. Positive log fold change in adjacent normal vs. CRC tumour indicates increased abundance in CRC tumour samples. Positive log fold change in healthy weight vs. overweight indicates increased abundance in healthy weight samples.

| **Sequence Variant Taxa** | **Log Fold Change (2 d.p)** | **Adjusted p value (2 s.f.)** |
| --- | --- | --- |
| **Adjacent Normal vs. CRC Tumour** | | |
| *Clostridium perfringens* | 3.88 | 0.029 |
| *Fusobacterium* | 5.33 | 0.014 |
| **Healthy Weight vs. Overweight** | | |
| Ruminococcaceae | 5.50 | 0.0025 |
| Ruminococcaceae | 2.60 | 0.042 |
| *Prevotella copri* | -3.38 | 0.042 |
| Erysipelotrichaceae | 4.23 | 0.035 |
| *Parvimonas* | -4.81 | 0.043 |
| *Bacteroides* | -3.41 | 0.0027 |
| Ruminococcaceae | 4.72 | 0.042 |
| Clostridiales | 5.10 | 0.022 |

**Supplementary Table 3. Average relative abundance of biomarkers identified by LEfSe analysis when comparing Healthy Weight and Overweight patient samples.**

| **Taxa** | **LDA Score** | **Healthy Weight Average % Abundance** | **Overweight Average % Abundance** |
| --- | --- | --- | --- |
| **Adjacent normal mucosa (CRC patients) Healthy Weight vs Overweight** | | | |
| Lactobacillus zeae | 4.14 | 0.01 | Not Detected |
| Tissierellaceae | 4.38 | 0.25 | 0.40 |
| Bacteroidetes | 4.41 | 30.97 | 46.14 |
| **CRC samples Healthy Weight vs. Overweight** | | | |
| Lactobacillaceae | 4.38 | 0.01 | <0.01 |
| Lactobacillus zeae | 3.83 | <0.01 | Not Detected |
| Acinetobacter | 3.86 | 0.01 | 0.01 |
